# 4566 The Impact of Migration on Viral Hepatitis Prevalence and Elimination Among 30 European Nations: A modeling approach

**DOI:** 10.1017/cts.2020.435

**Published:** 2020-07-29

**Authors:** Kenneth Valles, Andres Inzunza, Kritika Prasai, Lewis R Roberts

**Affiliations:** 1 Mayo Clinic College of Medicine and Science; 2 Tecnologico de Monterrey, School of Medicine; 3 Mayo Clinic Division of Gastroenterology and Hepatology; 4 Mayo Clinic

## Abstract

OBJECTIVES/GOALS: Hepatitis B and C virus causes inflammation of the liver and can lead to cirrhosis, liver failure, and hepatocellular carcinoma. The aim of this study is to generate a modeled estimate of changes in hepatitis B and C prevalence, and future sequelae, that accounts for recent mass migration to the European Union stemming from 50 high-emigration countries. METHODS/STUDY POPULATION: Total migrant population from 2013-2017 was obtained from the Eurostat population database. Demographics including country-of-origin, sex, and age distributions were used to determine migrant contributions to HBV and HCV prevalence where available. Undocumented migration estimates were obtained from the Institute of Migration database. Country-of-origin HBV and HCV prevalences were obtained for the select 50 country-of-origin nations from the Polaris Observatory and from systematic reviews. Disease progression was estimated using HBV and HCV outcome data for total populations from treatment guideline publication from the European Association for the Study of the Liver. RESULTS/ANTICIPATED RESULTS: Between 2013 and 2017, a total of 11,030,786 documented migrants born outside the EU arrived to the 30 nations. Germany, United Kingdom, and Spain received the greatest influx of persons and the majority of migration stemmed from countries in West Asia, the Middle East, and Africa. A significant proportion of total migration was driven by conflict-related crisis in Syria, and East and North Africa. The most significant increases in estimated total hepatitis case numbers, national prevalence increases, and future sequelae were seen in Germany and Sweden. DISCUSSION/SIGNIFICANCE OF IMPACT: Mass migration has significantly changed HBV and HCV disease burden in Europe over the past 5 years. Consequently, long-term outcomes of cirrhosis and HCC are also expected to increase. These increases are likely to disproportionally impact individuals of the migrant and refugee communities. HBV and HCV surveillance and management programs must strategically focus on individuals from high-burden age cohorts and nations. Screening and treatment would aid WHO elimination efforts while benefiting both the vulnerable individuals and host nations through reduction of morbidity, mortality, and associated healthcare expenses.

